# Inhibition of α-, β- and γ-carbonic anhydrases from the pathogenic bacterium *Vibrio cholerae* with aromatic sulphonamides and clinically licenced drugs – a joint docking/molecular dynamics study

**DOI:** 10.1080/14756366.2020.1862102

**Published:** 2021-01-20

**Authors:** Alessandro Bonardi, Alessio Nocentini, Sameh Mohamed Osman, Fatmah Ali Alasmary, Tahani Mazyad Almutairi, Dalal Saied Abdullah, Paola Gratteri, Claudiu T. Supuran

**Affiliations:** a Department NEUROFARBA – Pharmaceutical and Nutraceutical Section; Laboratory of Molecular Modeling Cheminformatics & QSAR, University of Firenze, Sesto Fiorentino, Italy; b Department NEUROFARBA – Pharmaceutical and Nutraceutical Section, University of Firenze, Sesto Fiorentino, Italy; c Chemistry Department, College of Science, King Saud University, Riyadh, Saudi Arabia

**Keywords:** Resistance, virulence, metalloenzyme, inhibition, MD

## Abstract

The binding mode of aromatic sulphonamides and clinically licenced drugs to the three carbonic anhydrase (CA, EC 4.2.1.1) isoforms from the human pathogen *V. cholerae* was here thouroghly characterised by a joint docking and molecular dynamics *in silico* protocol. In fact, VchCA, VchCAβ, and VchCAγ are crucial in the pathogen life cycle and growth and represent innovative targets to fight *V. cholerae* proliferation overcoming the spreading chemoresistance to the available drugs. A set of 40 sulphonamides/sulfamates VchCAs inhibitors was studied using the proteins homology built 3 D models unveiling the key and stable interactions responsible for a potent CA inhibition. This study has the aim to offer insights and guidelines for the future rational design of potent and selective inhibitors targeting CA isoforms from *V. cholerae* or other human pathogens.

## 1. Introduction

### 1.1. Cholera disease

*Vibrio* spp. are bacteria present in freshwater, estuarine and marine environments that prefer the warm and brackish water^1^. Among the ∼12 pathogenic species for humans of the >100 described *Vibrio* spp., *Vibrio cholerae* is the unique rod shape Gram-negative bacterium that provokes cholera, a disease endemic in low income countries^1^^,^^2^. Annually, cholera affects more than 2–4 million people worldwide with 21,000–143,000 deaths, half of them being children under 5 years old^3–5^. The infection occurs mainly by the faecal-oral route through contaminated food, or poorly sanitised water^6^^,^^7^, or through the person-to-person close contact^8^^,^^9^. *V. cholerae* enters in the gastrointestinal tract and reaches the small intestine^10^. Several intestinal environmental factors such as bicarbonate^11^, bile, unsaturated fatty acids, and reduced oxygen levels promote the co-transcription of toxin-co-regulated pilus (Tcp), cholera toxin (CT) and other colonization-associated genes (all encoded by regulon *toxT*), that allow the pathogen proliferation^12^. Using the filamentous surface appendage Tcp, *V. cholerae* is able to bind the same adjacent bacterial cells and to tightly adhere to enterocytes without disrupting the mucosal integrity^10^^,^^13^^,^^14^. Instead, the pathogen-secreted toxin CT, composed of two subunits *ctxA* and *ctxB*, recognises and binds the sialylated glycosphingolipid GM1 on the cytoplasmatic membrane of enterocytes with the pentameric *ctxB* subunit^15^. After endocytosis, CT enters in the endoplasmic reticulum (ER) via a retrograde transport, where the subunits are dissociated^15^. The enzymatic *ctxA* subunit released in the cytosol, upon allosteric activation by ADP ribosylation factor 6 (ARF6), is able to trigger the G-protein coupled receptor and consequently the adenylyl cyclase (AC)^15^. The high levels of produced cAMP stimulate the protein kinase A (PKA)-dependent phosphorylation of the cystic fibrosis transmembrane receptor (CFTR), responsible for the efflux of water and ions into the lumen of the small intestine, leading to diarrhea^15^. The profuse watery diarrhoea, together with vomiting and gastroenteritis, are the main clinical symptoms of cholera disease that, if untreated, results in death due to dehydration within 1–2 days^16^^,^^17^. To date, the long-term solutions to prevent cholera are the surveillance, sanitisation of the water, good hygiene practices, social mobilisation monitoring, and oral cholera vaccines^18^^,^^19^. On the other hand, the infection is treated by prompt administration of oral/intravenous rehydration solution (ORS)^20–22^, appropriate antibiotics (such as azithromycin and ciprofloxacin)^23^^,^^24^ and zinc^25^. While the administration of ORS is a fundamental but symptomatic therapy, the use of antibiotics is important to eradicate the cause of the illness. However, the spreading drug resistance to antimicrobial agents is threatening the efficacy of current chemotherapy, making the development of new antibiotic drugs with different mechanisms of action essential^26^.

### 1.2. *Vibrio cholerae* carbonic anhydrases

Bicarbonate is an important virulence factor for *V. cholerae* as it is a positive effector for *toxT* activity, promoting the transcription of genes that encode for Tcp, CT and other proteins implicated in proliferation^11^^,^^27–29^. These genes expression is significatively reduced by the addition of carbonic anhydrase inhibitors (CAIs)^27–29^. Thus, it is probable that *V.cholerae* uses the carbonic anhydrases (CAs, EC 4.2.1.1) system to accumulate bicarbonate into the cell for activating its virulence, as the bicarbonate levels are very high in the upper small intestine colonised by the pathogen and this bacterium does not encode bicarbonate transporter proteins in its genome^27–29^.

Such evidences make CAs interesting targets to prevent *V. cholerae* proliferation, offering the possibility to develop antibacterial drugs with an innovative mechanism of action to contrast the disease.

In detail, CAs are a superfamily of ubiquitous metalloenzymes, present in all life kingdoms, that catalyse the reversible hydration of carbon dioxide (CO_2_) into bicarbonate ion (HCO_3_^−^) and a proton^30^. To date, eight genetically unrelated families of CAs called α, β, γ, δ, η, ζ, θ and ι^31–38^ have been identified, but only α-, β-, γ- and ι-CAs are present in prokaryotes^28^^,^^39–43^_._ In microorganisms, these CAs are involved in photosynthesis (cyanobacteria), biosynthesis of amino acids, fatty acids, and nucleic acids, but also in proliferation, survival, and differentiation^44^.

The genome of *V. cholerae* encodes for three CAs, VchCA, VchCAβ, and VchCAγ respectively belonging to the α-, β- and γ-class. This suggests the important role of these enzymes in the pathogen physiology^28^^,^^44^^,^^45^.

VchCA (α-CA) consists of 239 amino acids, and shows a 30% identity with the two humans (h) α-CAs I and II. Moreover, VchCA maintains basic characteristics common to most α-CAs, that are the three histidine residues coordinating the zinc ion (H104, H106, and H123), a proton shuttle histidine residue (H79), and the gate-keeping glutamate-threonine dyad (E110, T189)^46^. No X-ray crystal structure of this enzyme is available so far.

The X-ray crystallography of VchCAβ (β-CA; PDB 5CXK)^47^ showed a tetrameric structure with four active sites, composed of monomers of 222 amino acids each^48–50^. Furthermore, VchCAβ preserves the common features of the β-class CAs that are the two cysteines and the histidine residue (C42, C101, and H98 from a same monomer) coordinated to the zinc ion, and the aspartate-arginine dyad (D44, and R46) responsible for the opening/closing of the active site^48–50^. In fact, β-CAs can exist in a type I (open active site) or type II (closed active site) enzyme, depending on the pH^48–50^. When the pH is < 8.0 the aspartate residue of the dyad coordinates the zinc ion as a fourth ligand in place of the water molecule/hydroxide ion, thus disabling the CO_2_ hydration reaction (type-II form). At pH > 8.0 the aspartate residue forms a salt bridge with the arginine of the dyad, allowing the zinc ion to be coordinated by a water molecule/hydroxide ion (type-I form)^48–50^. This pH regulation suggests that β-CAs activity is presumably regulated by the substrates and in particular by HCO_3_^-^ concentrations^48–50^. Hence, when *Vibrio colerae* reaches the upper small intestine, the high levels of bicarbonate can promote the type-II to type-I VchCAβ form conversion, assisting the virulence process.

VchCAγ is a trimeric enzyme with monomers formed by 184 amino acids (chains A, B and C) assembled to form three different active sites. As in the other γ-CAs, the zinc atom is coordinated by three conserved histidine residues (H65, and H94 from a chain and H89 from another chain) and a proton shuttle is present nearby (H68)^51^. As for the α-class CA from this organism, no X-ray crystal structure of the enzyme is available so far.

Kinetic parameters gathered in Table 1 show that VchCA (K_cat_ = 8.2 × 10^5^ s^−1^) is more active than VchCAγ (K_cat_ = 7.4 × 10^5^ s^−1^) and VchCAβ (K_cat_ = 3.3 × 10^5^ s^−1^), and all of them are more active than hCA I (K_cat_ = 2.0 × 10^5^ s^−1^)^45^^,^^52–55^.

**Table 1.** Kinetic parameters for the CO_2_ hydration reaction of α-CAs human cytosolic isozymes hCA I and II and VchCA measured at 20 °C and pH 7.5 in 10 mM HEPES buffer and 20 mM Na_2_SO_4_, and VchCAβ and VchCAγ measured at 20 °C, pH 8.3 in 20 mM TRIS buffer and 20 mM NaClO_4_^45^^,^^52–55^.

Enzyme	Species	Class	Activity level	K_cat_ (s^−1^)	K_M_ (M)	k_cat_/k_m_ M^−1^ x s^−1^	K_I_ AAZ (nM)
hCA I	Human	α	Moderate	2.0 × 10^5^	4.0 × 10^-3^	5.0 × 10^7^	250
hCA II	Human	α	Very high	1.4 × 10^6^	9.3 × 10^-3^	1.5 × 10^8^	12
VchCA	*V. cholerae*	α	Moderate	8.2 × 10^5^	11.7 × 10^-3^	5.4 × 10^7^	6.8
VchCAβ	*V. cholerae*	β	Moderate	3.3 × 10^5^	8.1 × 10^-3^	1.9 × 10^6^	4512
VchCAγ	*V. cholerae*	γ	Moderate	7.4 × 10^5^	11.5 × 10^-3^	6.4 × 10^7^	473

**Figure 1.**
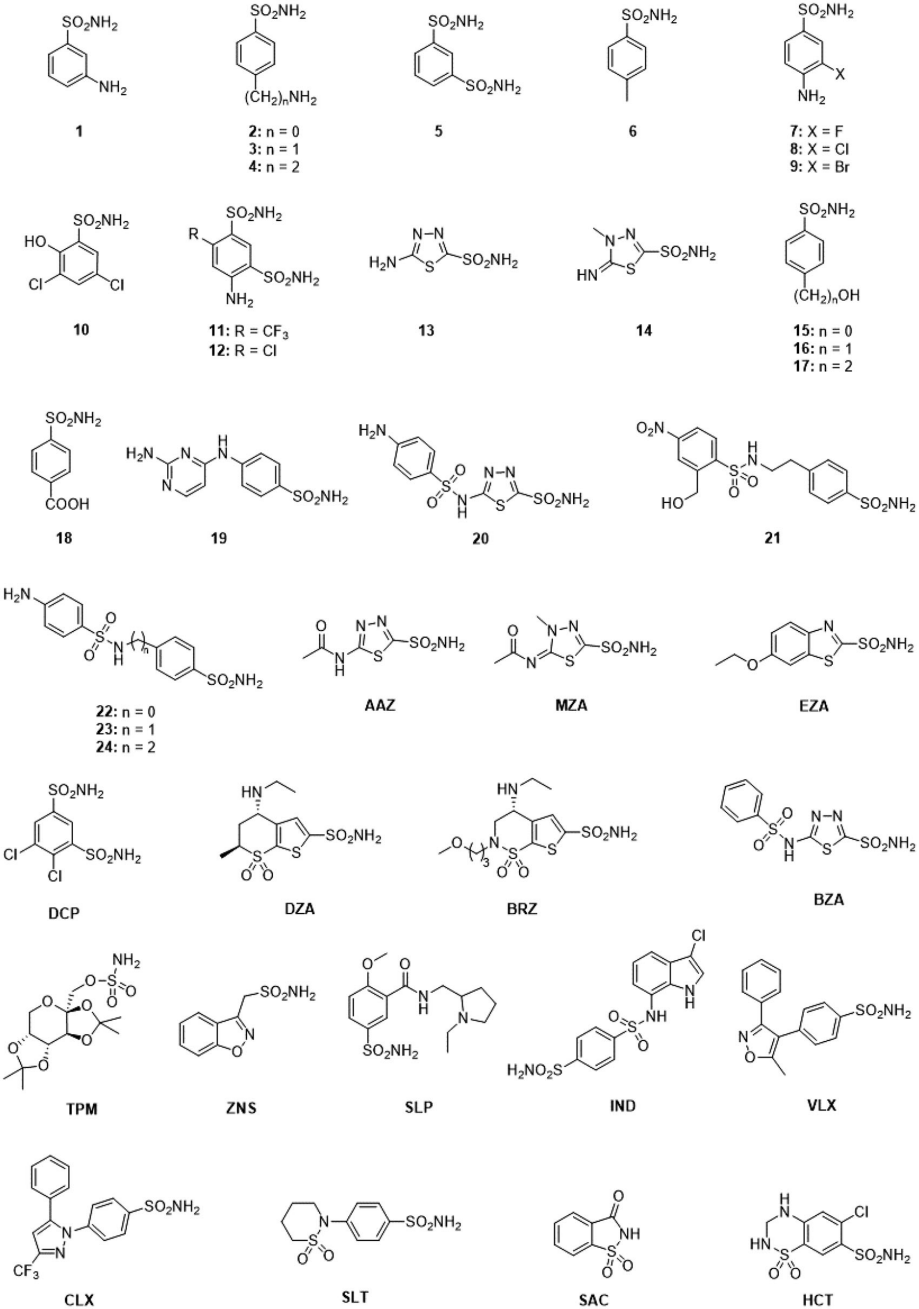
Structure of sulphonamides/sulfamates previously investigated as VchCAs inhibitors^45^.

Among many investigated chemotypes, primary sulphonamide derivatives stood out as the most potent CAIs to date^31^^,^^56^. Hence, a set of 38 primary aromatic/aliphatic sulphonamides (**1–24**, **AAZ**, **MZA**, **EZA**, **DCP**, **DZA**, **BRZ**, **BZA**, **ZNS**, **SLP**, **IND**, **VLX**, **CLX**, **SLT**, **HCT**), one secondary sulphonamides (**SAC**), and one sulphamate (**TPM**) shown in Figure 1, were evaluated for the inhibition of VchCA, VchCAβ and VchCAγ to characterise each isozyme response to inhibitors in search of new generation antiinfectives against *V. cholerae* (Table 2)^45^.

**Table 2.** Inhibition data of human isoforms hCA I and hCA II, and VchCA, VchCAβ and VchCAγ from *V. cholerae* with sulphonamides **1–24** and the clinically used drugs **AAZ-HCT** by a stopped-flow CO_2_ hydrase assay^45^.

	K_I_ (nM)^a,b^				
Cmpd	hCA I	hCA II	VchCA	VchCAβ	VchCAγ
**1**	45000	295	440	463	672
**2**	25000	240	471	447	95.3
**3**	25000	170	447	>10000	80.6
**4**	21000	160	402	>10000	69.0
**5**	28000	300	125	785	93.6
**6**	78500	320	219	>10000	76.3
**7**	8300	60	199	>10000	73.6
**8**	9800	110	139	9120	73.6
**9**	6500	40	133	>10000	95.3
**10**	7300	70	4656	>10000	544
**11**	5800	63	62.9	879	87.1
**12**	8400	75	45.3	4450	563
**13**	8600	60	23.5	68.1	66.2
**14**	9300	19	12.1	82.3	69.9
**15**	6	2	4.2	349	88.5
**16**	164	46	42.7	304	556
**17**	185	50	30.3	3530	6223
**18**	109	33	59.8	515	5100
**19**	95	30	4.7	2218	4153
**20**	690	12	0.59	859	5570
**21**	55	80	54.5	4430	764
**22**	21000	125	56.7	757	902
**23**	23000	133	71.5	817	273
**24**	24000	125	52.1	361	73.3
**AAZ**	250	12	6.8	4512	473
**MZA**	50	14	3.6	6260	494
**EZA**	25	8	0.69	6450	85.1
**DCP**	1200	38	37.1	2352	1230
**DZA**	50000	9	6.3	4728	87.3
**BRZ**	45000	3	2.5	845	93.0
**BZA**	15	9	4.2	846	77.6
**TPM**	250	10	>1000	874	68.8
**ZNS**	56	35	982	8570	725
**SLP**	1200	40	>1000	6245	77.9
**IND**	31	15	8.1	7700	91.3
**VLX**	54000	43	89.7	8200	817
**CLX**	50000	21	>1000	4165	834
**SLT**	374	9	88.4	455	464
**SAC**	18540	5959	>1000	275	550
**HCT**	328	290	79.5	87.0	500

^a^Errors in the range of 5–10% of the reported data, from 3 different assays. ^b^data from Di Fiore et al.^46^.

Many nanomolar inhibitors were identified against the three CAs, with efficacy against VchCA (K_I_s 0.59->1000 nM) > VchCAγ (K_I_s 66.2–6223 nM) > VchCAβ (K_I_s of 68.1->10000 nM)^45^. Here, a thorough interaction study was carried out *in silico* with derivatives in Figure 1 and the three VchCA isoforms.

## 2. Material and methods

### 2.1. Carbonic anhydrase inhibition

Results, discussion and methods of the CA inhibition assay for compounds **1–24** and used drugs **AAZ-HCT** were previously reported^45^.

### 2.2. Molecular modelling

The homology models of VchCAα, VchCAβ and VchCAγ^57^ were prepared using the Protein Preparation Wizard tool implemented in the Schrödinger suite^58^. The energy minimisation protocol with a root mean square deviation (RMSD) value of 0.30 Å was applied using force field OPLS3e. The ligand structures were prepared by Maestro^58^b and evaluated for their ionisation states at pH 7.4 ± 0.5 with Epik^58^c. The conjugate gradient method in Macromodel^58^^e^ was used for energy minimisation (maximum iteration number: 2500; convergence criterion: 0.05 kcal mol^−1 ^Å^−1^). The software Glide was used for docking^58^^f^. Grids were centred on the centroids of the zinc-coordinating residues and ligands were docked using standard precision mode (SP). The best pose of a subset of compounds to the three VchCAs, evaluated in terms of anchorage, hydrogen bond interactions and hydrophobic contacts, was submitted to a MD simulation using Desmond and the OPL3e force field^58^^g,^^59^. Specifically, the system was solvated in an orthorhombic box using TIP4PEW water molecules, extended 15 Å away from any protein atom. It was neutralised adding chlorine and sodium ions. The simulation protocol included a starting relaxation step followed by a final production phase of 100 ns. In particular, the relaxation step comprised the following: (a) a stage of 100 ps at 10 K retaining the harmonic restraints on the solute heavy atoms (force constant of 50.0 kcal mol^−1 ^Å^−2^) using the NPT ensemble with Brownian dynamics; (b) a stage of 12 ps at 10 K with harmonic restraints on the solute heavy atoms (force constant of 50.0 kcal mol^−1 ^Å^−2^), using the NVT ensemble and Berendsen thermostat; (c) a stage of 12 ps at 10 K and 1 atm, retaining the harmonic restraints and using the NPT ensemble and Berendsen thermostat and barostat; (f) a stage of 12 ps at 300 K and 1 atm, retaining the harmonic restraints and using the NPT ensemble and Berendsen thermostat and barostat; (g) a final 24 ps stage at 300 K and 1 atm without harmonic restraints, using the NPT Berendsen thermostat and barostat. The final production phase of MD was run using a canonical NPT Berendsen ensemble at temperature 300 K. During the MD simulation, a time step of 2 fs was used while constraining the bond lengths of hydrogen atoms with the M-SHAKE algorithm. The atomic coordinates of the system were saved every 100 ps along the MD trajectory. Protein and ligand RMSD values, ligand torsions evolution and occupancy of intermolecular hydrogen bonds and hydrophobic contacts were computed along the production phase of the MD simulation with the Simulation Interaction Diagram tools implemented in Maestro.

## 3. Docking and molecular dynamics

For an exhaustive comprehension of the inhibition profile of compounds **1–24**, **AAZ**, **MZA**, **EZA**, **DCP**, **DZA**, **BRZ**, **BZA**, **ZNS**, **TPM**, **SLP**, **IND**, **VLX**, **CLX**, **SLT**, **SAC**, **HCT** and to understand the key interactions for their recognitions within VchCA, VchCAβ, and VchCAγ, an *in silico* investigation was carried out applying a computational protocol that includes docking studies, MM-GBSA refinements, and MD calculations.

Sulphonamides and bioisosteres (i.e. sulfamates and sulfamides) act as zinc-binders CAIs against the human^56^^,^^61^ and bacterial^62^ α-CAs, binding the zinc ion by the deprotonated nitrogen atom (SO_2_NH^-^) according to a tetra-coordinated geometry around the metal ion. Aromatic compounds such as benezenesulfonamides and 1,3,4-thiadiazole-2-sulphonamides show a better inhibitory activity than aliphatic sulphonamide derivatives as their binding mode is stabilised by vdW contacts involving conserved lipophilic residues in the inner active site (namely L198, V121, V143 in hCA II) and the aromatic portion bearing the zinc-binding group.

X-ray studies on β-CAs pointed out that aromatic sulphonamides and sulfamides occupied the fourth coordination site site around the zinc ion that in β-CAs is in a pseudotetrahedral coordination environment. Indeed, the deprotonated nitrogen atom (SO_2_NH^-^) is coordinated to the Zn and the aromatic ring is stabilised by a π-π stacking interaction with the side chain of an aromatic residue nearby (i.e. phenylalanine or tyrosine)^63^^,^^64^. Instead no X-ray crystallography data exist which show the binding mode of sulphonamide to γ-CAs. However, it is reasonable to assume that primary sulphonamide derivatives act as fourth ligand of the zinc coordination sphere replacing the water molecule also in γ-CAs. Interestingly, in a recent modelling study performed on sulphonamide inhibitors against the β- and γ-CA isoforms from *E. faecium*^65^, the inhibitors were predicted to act as metal binders against the γ-CA class adopting both a tetrahedral and pentameric coordination.

With the exception for the closed, type II, form of β-CA from *Vibrio cholerae*, to date in the PDB^66^ there are no solved structures for VchCAα,VchCAγ and type I/open VchCAβ, both in apo form and or in complex with ligands. In this study, the homology-built models of the three classes of *Vibrio cholerae* CAs, already obtained for a previous investigation, were used to shed light on the binding mode of sulphonamide derivatives to all CA classes of from this pathogen. First, the ligands were docked within the binding cavity of the three CAs, then the stability of the binding poses was assessed by molecular dynamic simulations. The results are presented according to each enzyme isoform.

### 3.1. Vchca

All docking solutions for compounds in Figure 1 localise the ligands at the bottom of the conical cavity of the enzyme. Here the SO_2_NH- moiety coordinated around the zinc ion according to a tetrahedral geometry (Figure S1, Supporting Information). Moreover, the sulphonamide NH- and S=O groups are in H-bond contact with the side chain hydroxyl group of T189 (N^−^⋯H-O) and with the backbone of the same residue (O…H-N) respectively. The binding orientations of both the benzenesulphonamide and the 1,3,4-thiadiazole-2-sulphonamide inhibitors are stabilised by van der Waals (vdW) interactions that occur between the phenyl ring and the hydrophobic residues L188, V135, and V125 (Figure S1A). In addition 1,3,4-thiadiazole-2-sulphonamides form an H-bond by the N3 atom of the heterocycle with T190 OH group (Figure S1B), which might contribute to the generally increased inhibition profile shown by these derivatives compared to the benzenesulphonamides.

**Figure 2.**
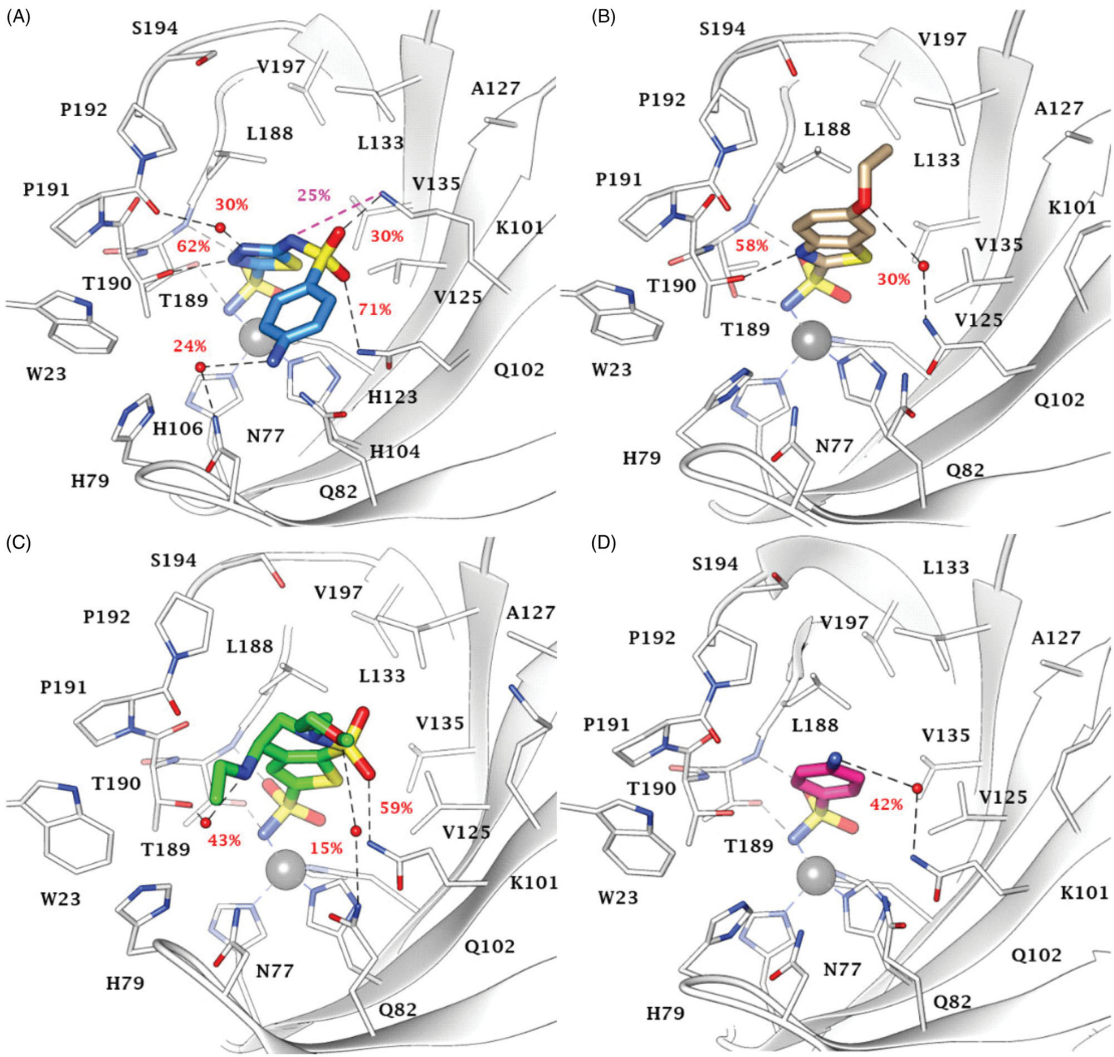
Most populated binding conformation along the MD trajectory for (A) **20** (blue), (B) **EZA** (tan), (C) **BRZ** (green), and (D) **2** (pink) within VchCA active site. H-bonds and salt bridge interactions are depicted as black and magenta dashed lines, respectively. The occupancy over the MD simulation of interactions not involving the zinc-binding group is indicated as percentage.

100 ns long MD simulations were carried out to monitor the structural flexibility of the poses. Derivatives **20** (K_I_ = 0.59 nM), **EZA** (K_I_ = 0.69 nM), and **BRZ** (K_I_ = 2.5 nM) were considered as representatives among the compounds in Figure 1 having the best inhibition profiles, while **2** (K_I_ = 471 nM) was chosen due to its worst inhibition profile (Figure 2). Noteworthy, all the bound poses from docking totally maintained the coordination the zinc ion and the H-bond network with T189 for more than 96% of the MD (Figure 2). In addition, H-bonds involving the N3 atom of the 1,3,4-thiadiazole **20** and 1,3-benzo[d]thiazole **EZA** ring persist for the 62%, and 58% of the simulation course (Figures 2(A,B)). The sulphonamide linker in **20** is involved in a H-bond network with the carboxyamide and the protonated amino groups in the side chains of Q102 and K101 respectively (Figure 2(A)). Moreover, due to the net negative charge of the linker sulphonamide at the physiological pH, **20** forms a salt bridge interaction with K101, while further contributes to the stabilisation of the pose come from the water bridged H-bonds that occur between the Q82 side chain and the NH_2_ group and the heterocyclic N4 and the P191 carbonyl group (hetN4⋯HO-H⋯O = C-P191; Figure 2(A)).

Water bridged H-bonds also occur for **EZA** (Figure 2(B)) and **BRZ** (N-H…O-H T190; H3C-O…H-N Q82; Figure 2(C)). The ligands are further stabilised by a wide ensemble of interactions, mainly 15–55% stable vdW interactions involving L188, P192, L133, W23 (Figure 2(B,C)). For 59% of the MD simulation, the endocyclic **BRZ** sulphonamide S=O is engaged in an H-bond interaction with the Q102 side chain NH2.

The MD study of sulphanilamide **2** highlighted 65% stable vdW interactions with L188, and water bridged H-bonds that the outer NH_2_ can stably form with hydrophilic active site residues, such as Q102 (Figure 2(D)).

Outcomes from MD computation may also provide insights for understanding the inhibition profile of other benzenesulfonamides **1**, **3**, **4**, **6–9**, **19**, **21**, and **23** (Figures S2-S3, Supporting Information). Similarly to derivative **2**, the amine function of **1** (K_I_ = 440 nM), **3** (K_I_ = 447 nM) and **4** (K_I_ = 402 nM) is oriented towards the hydrophilic half of the active site, not able to form direct H-bonds with the protein (Figure S2A-B). Instead, the substitution of the aromatic amine of **2** with a methyl moiety to give compound **6** (K_I_ = 219 nM) increased the inhibition profile, probably because of the increased vdW interactions with P191 and P192 (Figure S2A). The halogenation of sulphanilamide **2** in position 3 with a fluorine, chlorine, or bromine atom to give compound **7–9** (K_I_ = 133–199 nM) enhances the hydrophobic contacts with the lipophilic half of the active site in the order Br > Cl > F (Figure S2C). The elongation of the tail of sulphanilamide **2** by a 2-amino-pyrimidin-4-yl in **19** (K_I_ = 4.7 nM) allows the outer amine group to form direct H-bonds, namely with the C=O of P191, that together with other probable water-mediated interactions could improve the binding to the target (Figure S2D). Likewise, the hydroxymethyl group of derivative **21** (K_I_ = 54.5 nM) engages an H-bond with Q82 side chain NH_2_ (Figure S3A). The binding mode of compound **23** (K_I_ = 71.5 nM) is shown in Figure S3B as representative of the set **22–24** (K_I_ = 52.1–71.5 nM). The sulphonamide linker S = O group receives an H-bond by the NH_2_ of Q102 side chain. Residue K101 may be involved in the interaction with the ligand, that is, an H-bond with the S=O group for compounds **22–24**, and a salt bridge interaction with the N^-^ moiety of compound **22**). Derivatives **13** (K_I_ = 23.5 nM) and **14** (K_I_ = 12.1 nM) are better inhibitors than sulphanilamide **2** probably because of the three H-bonds with T189 and T190 stabilising the scaffold of 1,3,4-thiadiazole-2-sulphonamide derivatives (Figure S3C). The presence of a N4-methyl group in derivative **14** enhances the hydrophobic contacts with the lipophilic half of the active site (P191, P192, and L188), justifying the twofold inhibition profile as compared to that one of **13**. Moreover, the clinically used acetylated compounds **AAZ** (K_I_ = 6.8 nM) and **MZA** (K_I_ = 3.6 nM) are 4-fold more active than **13** and **14**, owing to additional H-bond that the acetyl C = O moiety can receive from the NH_2_ of Q102 side chain (Figure S3D). Similarly to its precursor **13**, **BZA** (K_I_ = 4.2 nM), showed an H-bond between the ligand S = O group and Q102 (Figure S4A). **DZA** (K_I_ = 6.3 nM) adopted a similar binding mode as the structurally resembling **BRZ** (Figure 2C), whereby the NH_2_^+^ moiety is involved in H-bond with P191the carbonyl group C = O and the endocyclic sulphonamide engages polar contacts with the hydrophilic half (Figure S4B). **IND** (K_I_ = 8.1 nM), uniquely having a reversed –SO_2_NH- linker, showed a binding orientation in which the sulphonamide NH moiety donates an H-bond to the P191 backbone C=O and the indolic ring is stabilised by a π-π stacking with the indole of the W23 (Figure S4C). All poses computed for **SLP** feature strains occurring in the contact between the ligand pyrrolidine and Q102, applicable to explain a K_I_ value above 1000 nM (Figure S4D).

### 3.2. Vchcaβ

The binding site of VchCAβ is narrower than those of VchCA and VchCAγ. This made the binding mode prediction more challenging and experimentally led to the generally less favourable inhibition profile of all compounds against this isoform (Table 2). According to literature, the SO_2_NH^−^ was found as coordinated around the zinc ion at the dimeric interface, engaging H-bonds with the OH group of Y83, NH_2_ of Q33 side chain, and the carboxylic function of D44 (Figure S5, Supporting Information). Moreover, the aromatic ring bearing the zinc-binding group (benzene/1,3,4-thiadiazole-2-sulphonamide) is stabilised by a π-π interaction with Y83 aromatic ring and by vdW contacts with V59.

**Figure 3.**
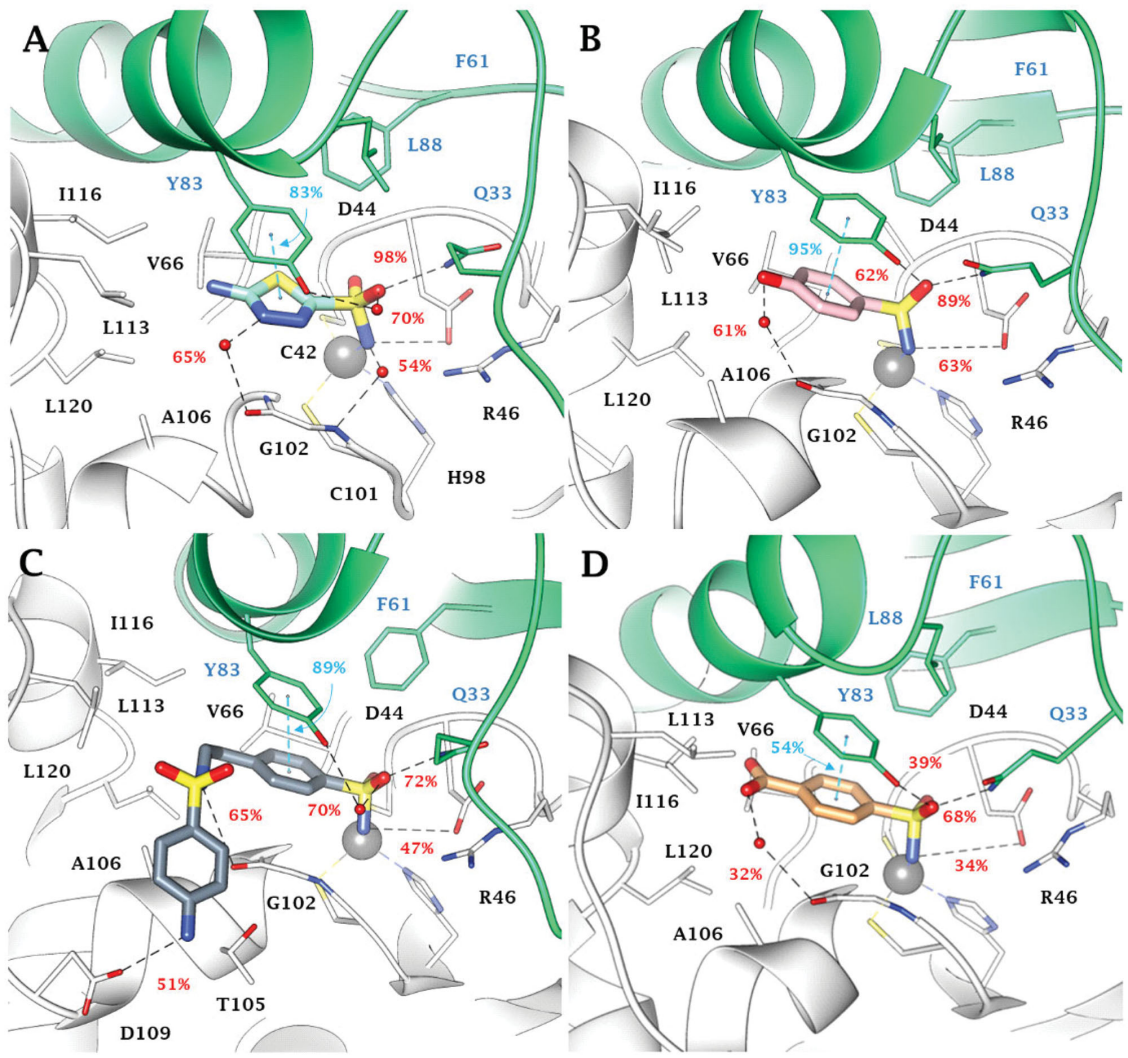
Most populated binding conformation along the MD trajectory for (A) **13** (aquamarine), (B) **15** (pink), (C) **24** (grey), and (D) **18** (orange) within VchCAβ active site. H-bonds and π-π stacking interactions are depicted as black and cyan dashed lines, respectively. The occupancy over the MD simulation of interactions not involving the zinc-binding group is indicated as percentage.

MD simulations performed on **13** (K_I_ = 68.1 nM), the best VchCAβ inhibitor in Figure 1, and on three compounds with medium inhibition profile **15** (K_I_ = 349 nM), **24** (K_I_ = 361 nM), and **18** (K_I_ = 515 nM) confirmed the total permanency of the metal coordination and the high stability of the interaction network involving the aromatic sulphonamide core and the aminoacidic residues D44, Q33, Y83 and V59, with which the SO_2_NH^-^ moiety is both in H-bond contacts (D44 and Q33) and forms π-π stacking (Y83) and vdW interactions (V59: stable for 24–93% of the MD; Figure 3).

Additionally, the binding orientation of **13** is firmly held within the binding site by water bridged H-bonds involving G102. It is likely that these interactions contribute to make the positioning of thiadiazole derivatives witin the binding site more stable as compared to the benzenesulfonamides (Figure 3(A)). Among the latter, the phenol derivative **15** also forms a water-mediated H-bond with G102 (Figure 3(B)).

The short active site of VchCAβ obliges inhibitor **24** to fold at the tail level; as a result the outer amine group holds for 51% of the MD course H-bond distance with D109 carboxylic function, while sulphonamide linker NH forms an H-bond with the C = O group of G102 (Figure 3(C)). Moreover, hydrophobic contacts persist with P111, L113, I116 and A106 for 16–30% of the MD course.

Similarly to **15**, compound **18** forms a water bridged H-bond with G102 C=O by the *p*-carboxy function (Figure 3(D)); however, the presence of the charged group COO^-^ nearby the lipophilic area of the active site might be the cause of the weakening of the binding interaction up to a K_I_ value of 515 nM. In the case of **16** (K_I_ = 304 nM), and **17** (K_I_ = 3530 nM), the chain elongation is effective only for *n* = 1 (**16**), while in the alkylamine analogues **3** and **4** (with K_I_ > 10000 nM) the presence of some ligand strains, allowed by the docking algorithm, together with the proximity of the charged amine group to the lipophilic part of VchCAβ active site, is responsible for the decreasing in the inhibition profile. Steric hindrance effects prevent the complementarity of the compounds **10** and **11** with the target although the second sulphonamide group in **11** directly binds with the hydroxyl side chain group of Y83 through an H-bond interaction. Modelling the flexible positioning of **13** and **14** (K_I_ = 82.3 nM) within pointed out the detrimental effect of the N4-methylation of the thiadiazole ring, as it disables the water-bridged H-bond stabilisation observed with **13** (Figure S7A). Further, N-acetylation of **13** and **14** to give derivatives **AAZ** (Figure S7B) and **MZA** (Figure S7C) produces steric strains within the VchCAβ active site that cause a drop of inhibitory efficacy. Similarly, it was observed for **EZA** (Figure S7D).

### 3.3. Vchcaγ

Within the cylindrical active site of VchCAγ all sulphonamide inhibitors bind the zinc ion according to a tetrahedral geometry (Figure S8, Supporting Information). Here, both the NH^-^ and S=O groups of the sulphonamide moiety engage H-bonds with the C=O and NH_2_ moieties of Q59 side chain, respectively, with the NH^-^ group also acting as an acceptor of an H-bond by the OH of Y159.

**Figure 4.**
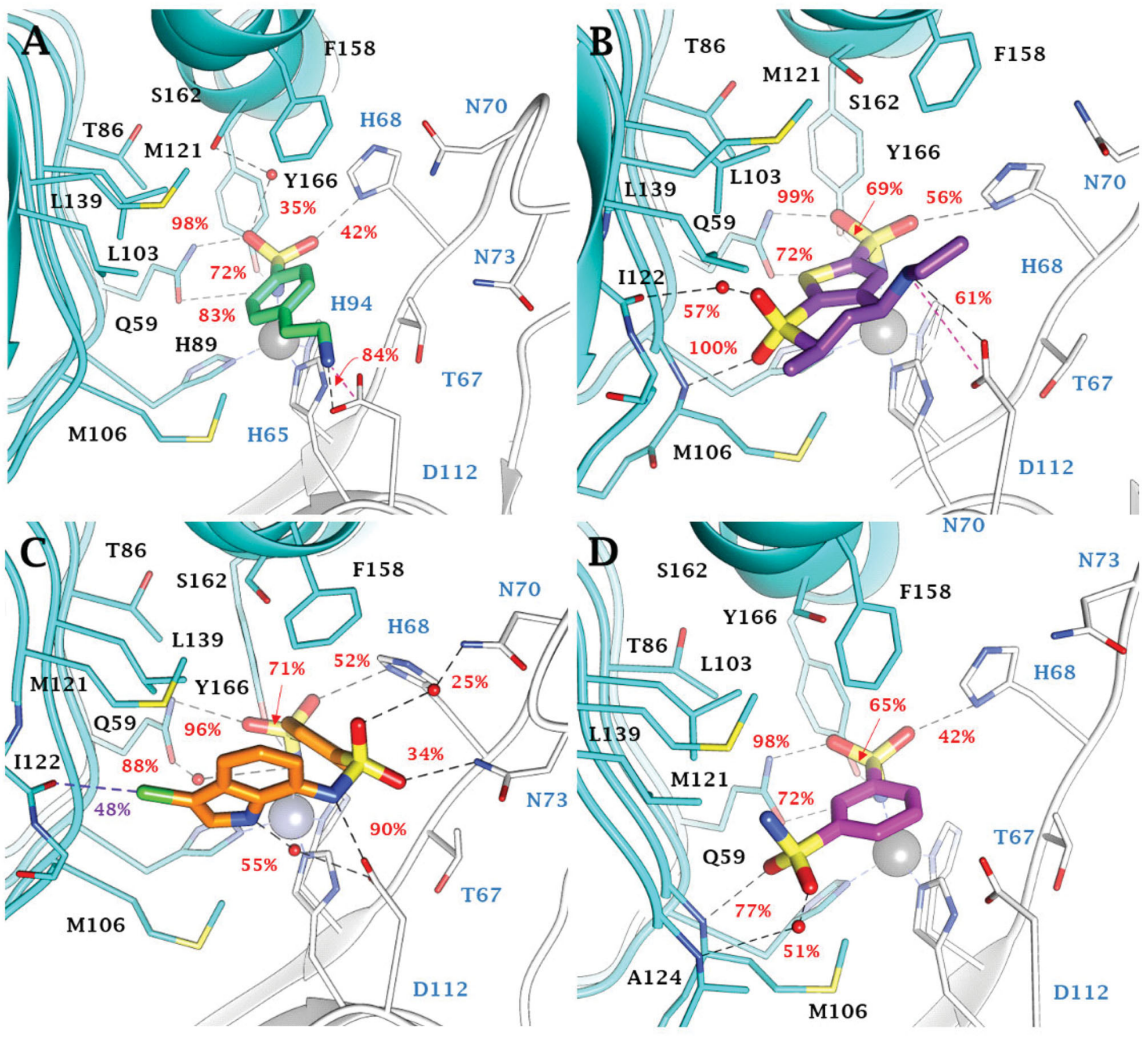
Most populated binding conformation along the MD trajectory for (A) **4** (spring green), (B) **DZA** (forest green), (C) **IND** (yellow), and (D) **5** (orchid) within VchCAγ active site. H-bonds, salt bridge, π-π stacking interactions, and halogen bonds are depicted as black, magenta, cyan, and green dashed lines, respectively. The occupancy over the MD simulation of interactions not involving the zinc-binding group is indicated as percentage.

No significant differences are observed in the inhibition profile of 1,3,4-thiadiazole and benzenesulfonamide inhibitors in VchCAγ. MD simulations performed on some of the most effective inhibitors, **4** (K_I_ = 69.0 nM), **DZA** (K_I_ = 87.3 nM), **IND** (K_I_ = 91.3 nM), and **5** (K_I_ = 93.6 nM) (Figure 4) pointed out the key role of H68 in the stabilisation of aromatic (benzene/1,3,4-thiadiazole) sulphonamides through H-bond and π-π stacking interactions. The analysis of the trajectories unequivocally demonstrates the stability of these interactions which are maintained for most of the simulation. In addition, both water molecules and salt bridges occurring between charged groups of the ligands and the target also play a role in the stabilisation of the ligand-target conformations. These are the case for compounds **4** (SO…H-OH…HO S162, NH3+…-OOC D112), **DZA** (-NH2+…-OOC D112, SO…H-OH…O = C I122, SO…H-N M106) and the clinical inhibitor **IND** (-NH2+…-OOC D112, SO…H-OH…H-N N70, SO…H-N N73).

Moreover, in this latter, the sulphonamide linker and indole NH groups form direct or water bridged H-bonds with the carboxylic group of the D112 (Figure 4(C)). A 48% stable halogen bond is also established by the ligand chlorine atom with the C=O group of I122 backbone. Also the sulphonamide linker S=O groups of derivative **5** are implicated in the binding to the protein, i.e. forming direct or water bridge H-bonds with the backbone NH of M106 and A124 (Figure 4(D)).

Interesting flexible ligand docking results were derived also for compounds not investigated by MD. The outcomes from docking allow to shed light on the effects of the substitution position as well as the chain elongation and the linker length. The better inhibition profile of **2** (K_I_ = 95.3 nM) compared to **1** (K_I_ = 672 nM, Figure S9A) arose from the failure for **1** to establish H-bond interaction with the side chain carboxylic group of D112.

Inhibitor **3** showed a similar binding mode with its homolog **4** (Figure 4(A)), with a charged H-bond forming between the ligand NH_3_^+^ and the D112 carboxylic moieties (Figure S9B). The substitution in *para* position of derivatives **6–9** facilitates vdW contacts with the enzymatic counterpart and, as a result, inhibition profiles with respect to the leads (Figures S9C-D).

The 1,3,4-thiadiazole-2-sulphonamides **13** (K_I_ = 59.2 nM) and **14** (K_I_ = 69.9 nM), potent VchCAγ inhibitors, formed H-bonds between the amine group of the ligands and the COO^-^ of D112 (Figure S10A). On the contrary, this contact is prevented by the steric hindrance of the acetyl pendant in **AAZ** (K_I_ = 473 nM) and **MZA** (K_I_ = 494 nM, Figure S10B). Notably, the elongation of the aliphatic chain in derivatives **15**–**17** (K_I_ = 88.5 − 556-6223 nM) increasingly prevents the stability of the H-bond between the OH in the hydroxyl alkyl chain and the D112 carboxylic moiety (Figure S10C).

Compound **11** showed a similar network of interactions as **5** observed so far but, more than this, further vdW interactions between the CF_3_ substituent and T67 side chain increase its inhibitory efficacy. The formation of H-bond contacts together with polar interactions of the outer amine group and the neighbour residues, resulted in **24** (K_I_ = 87.1 nM) > **23** (K_I_ = 273 nM) > **22** (K_I_ = 902 nM)

As a result of this *in silico* analysis, it can be pointed out that heteroaromatic sulphonamide CAIs show greater VchCA and VchCAβ inhibition than benzenesulphonamides, as a result of additional direct or water-mediated H-bonds engaged by the N atoms on the heterocycle with T189 and P191 in VchCA and G102 in VchCAβ. In contrast, no analogue stabilisation can occur in the wider VchCAγ active site providing insights about the comparable VchCAγ inhibitory profiles shown by these two types of aromatic sulphonamides.

The joint docking/MD study also suggests that small, not unwieldy CAIs (e.g. **13** and **14**) can more efficiently accommodate and bind in the narrow VchCAβ active site, inducing a greater inhibition than bulky derivatives. Further, it was shown that derivatives able to attain and interact with D109 through H-bonds (e.g. **24**) showed an increased VchCAβ inhibition potency. Similarly, residue D122 in VchCAγ was identified as a key residue for H-bond/salt bridge interactions for increasing the binding stability and inhibition of the γ-class isozyme.

## 4. Conclusions

*V. cholerae* encodes for three CAs (VchCA, VchCAβ, and VchCAγ) that are crucial in the pathogen life cycle and growth. These enzymes are interesting targets to prevent *V. cholerae* proliferation. They offer the possibility to develop antibacterial drugs with an innovative mechanism of action for overcoming the spreading chemoresistance to the available drugs. A set of 40 aromatic sulphonamides and clinically licenced drugs (shown in Figure 1) were previously evaluated for the inhibition of VchCA, VchCAβ and VchCAγ to characterise each isozyme response to inhibitors in search of a new generation antiinfectives for the treatment of the disease. For the first time, this extended panel of CA inhibition profiles was here characterised at the molecular level by a thorough *in silico* study to point the structural parameters featuring each isozyme inhibition. Using the homology built 3 D structure of the three VchCAs, a joint docking and MD protocol was adopted to unveil the key and stable interactions responsible for a potent CA inhibition. This study might offer insights and be of crucial relevance in the rational design of new potent and selective inhibitors targeting CA isoforms from *V. cholerae* or other human pathogens.

## Supplementary Material

Supplemental Material
