# Supplementary material for: Inhibition of α-, β- and γ-carbonic anhydrases from the pathogenic bacterium Vibrio cholerae with aromatic sulphonamides and clinically licenced drugs – a joint docking/molecular dynamics study
Source: J Enzyme Inhib Med Chem. 2021 Jan 20;36(1):469–79. doi: 10.1080/14756366.2020.1862102 (PMC7822066; doi:10.1080/14756366.2020.1862102)
Supplement: Supplemental Material [file IENZ_A_1862102_SM2996.pdf]

Supporting Information

Inhibition of α-, β- and γ-carbonic anhydrases from the pathogenic bacterium *Vibrio cholerae* with aromatic sulfonamides and clinically licensed drugs – a joint docking/molecular dynamics study

Alessandro Bonardi, Alessio Nocentini, Sameh Mohamed Osman,* Fatmah Ali Alasmary, Tahani Mazyad Almutairi, Dalal Saied Abdullah, Paola Gratteri,* Claudiu T. Supuran

Docking studies with VchCA S2

Docking studies with VchCAβ S5

Docking studies with VchCAγ S7

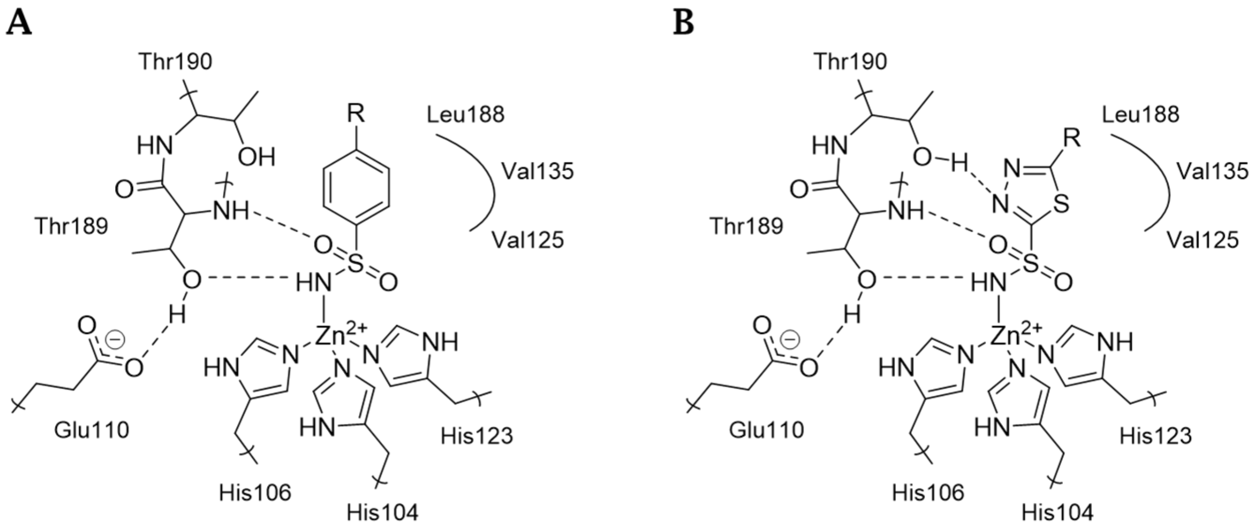

Figure S1. 2D representation of residues involved in the stabilization of (A) benzenesulfonamide and (B) 1,3,4-thiadiazole-2-sulfonamide inhibitors in the VchCA active site.

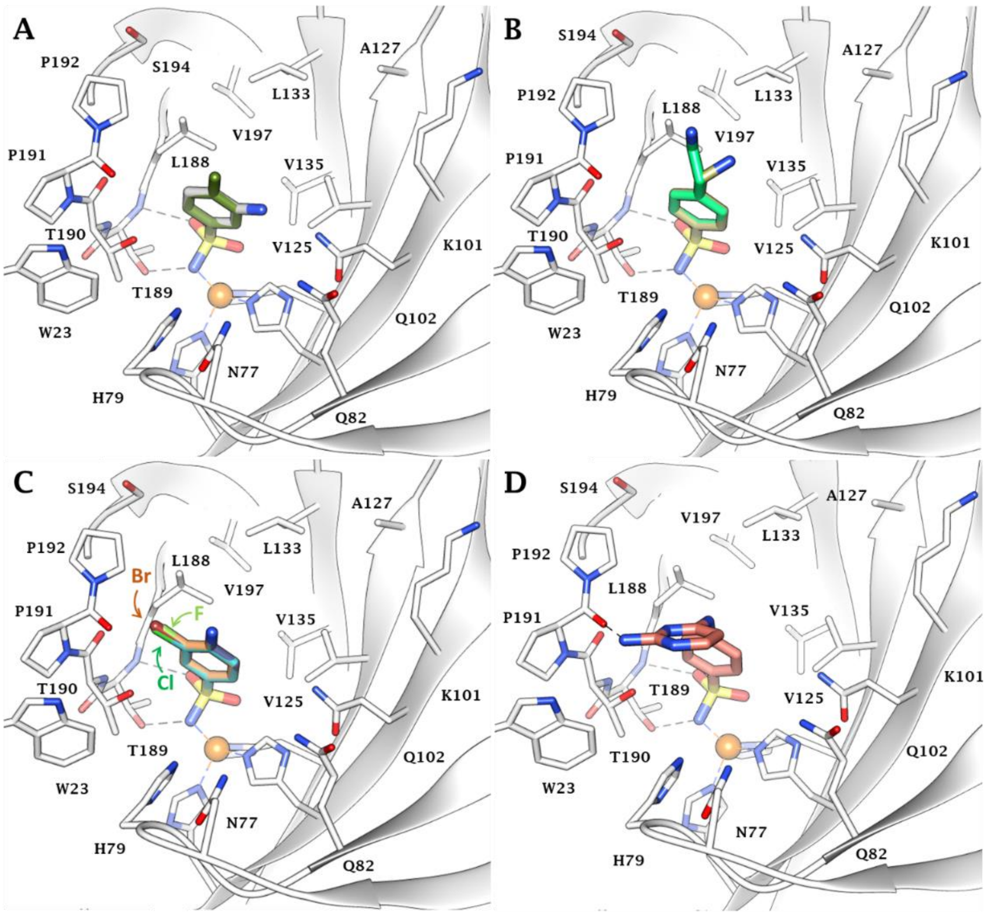

Figure S2. Predicted binding mode of ligands (A) **1** (light gray), and **6** (olive drab), (B) **3** (dark khaki), and **4** (spring green), (C) **7** (orange), **8** (cyan), and **9** (purple), (D) **19** (hot pink) within VchCA active site. H-bonds are depicted as black dashed lines.

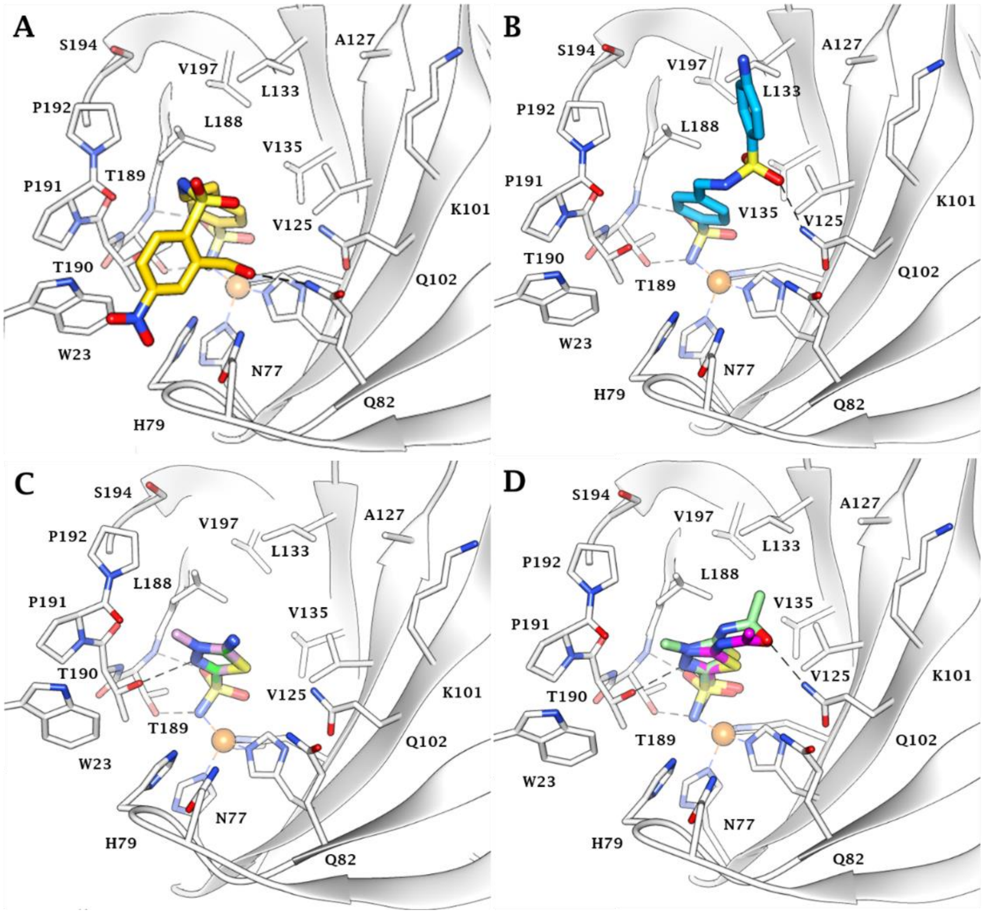

Figure S3. Predicted binding mode of ligands (A) **21** (gold), (B) **23** (deep sky blue), (C) **13** (green), and **14** (plum), (D) **AAZ** (magenta), and **MZA** (light green) within VchCA active site. H-bonds are depicted as black dashed lines.

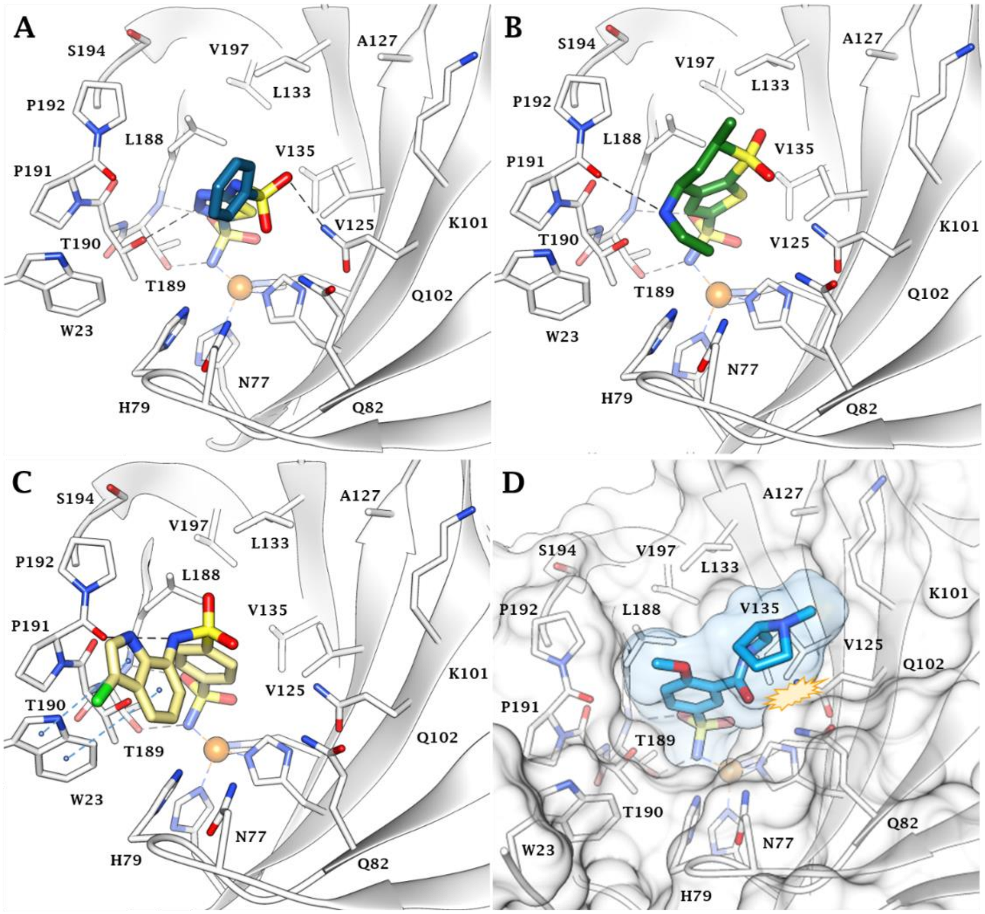

Figure S4. Predicted binding mode of ligands (A) **BZA** (blue), (B) **DZA** (forest green), (C) **IND** (khaki), and (D) **SLP** (dodger blue) within VchCA active site. H-bonds, and π-π stacking interactions are depicted as black, and cyan dashed lines, respectively. Clash contacts between the ligand-target surfaces are represented as orange clouds.

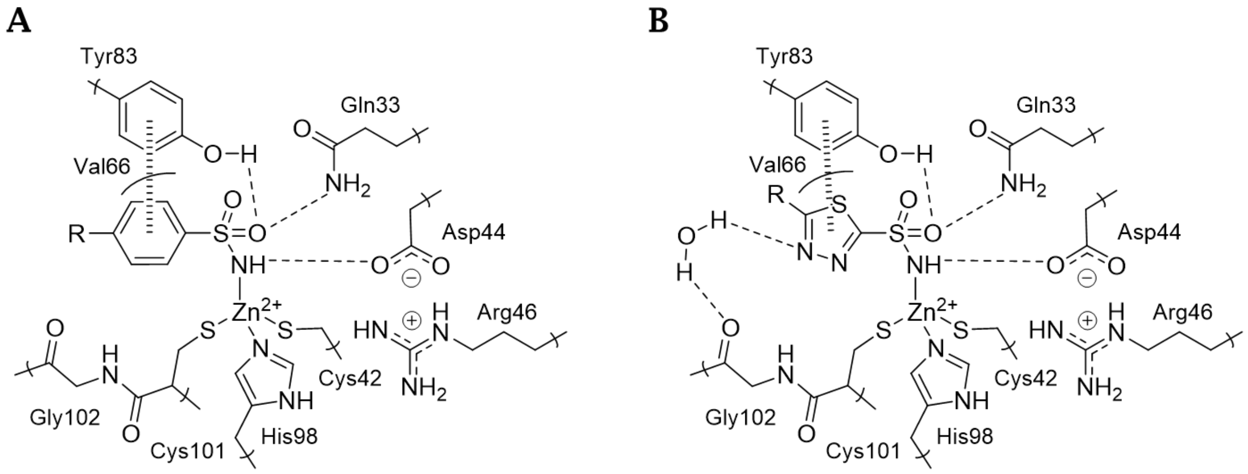

Figure S5. 2D representation of residues involved in the stabilization of (A) benzenesulfonamide and (B) 1,3,4-thiadiazole-2-sulfonamide inhibitors in the VchCAβ active site.

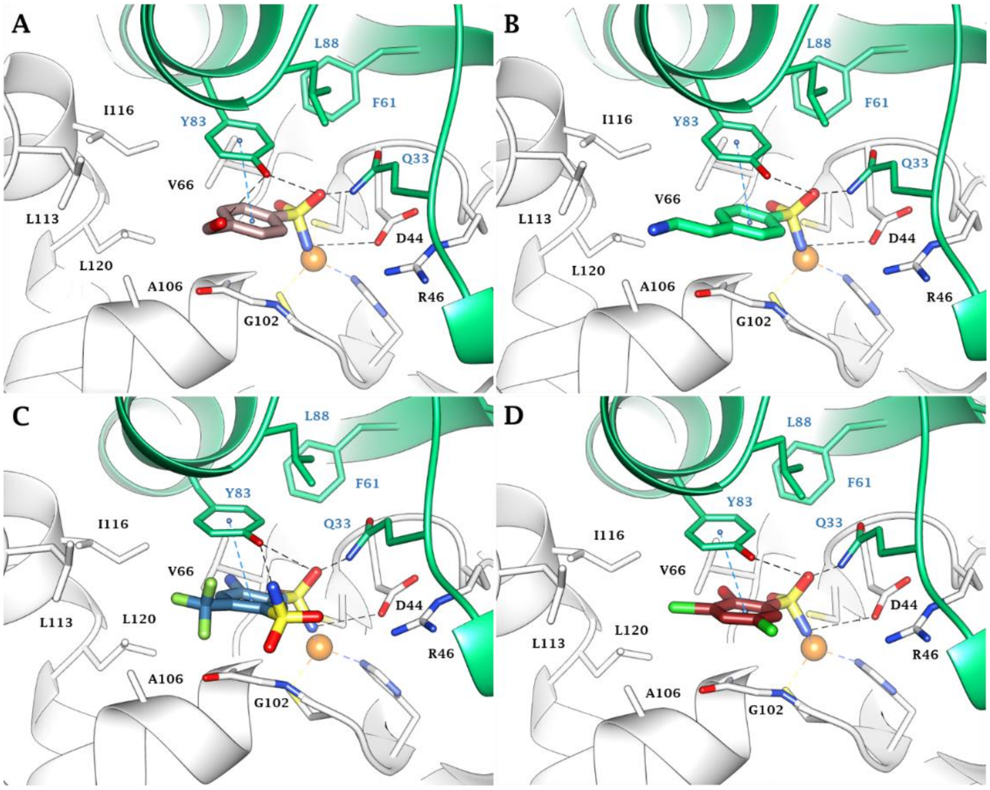

Figure S6. Predicted binding mode of ligands (A) **16** (rosy brown), (B) **4** (spring green), (C) **11** (steel blue), and (D) **10** (firebrick) within VchCAβ active site. H-bonds, and π-π stacking interactions are depicted as black, and cyan dashed lines, respectively.

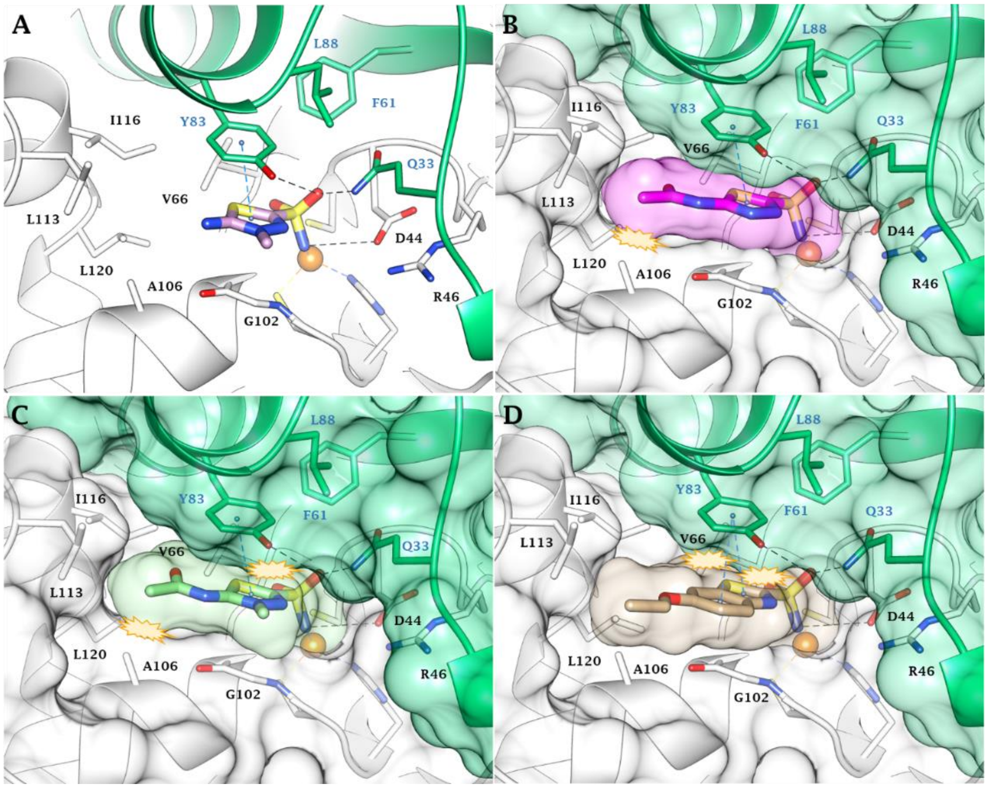

Figure S7. Predicted binding mode of ligands (A) **14** (plum), (B) **AAZ** (magenta), (C) **MZA** (light green), and (D) **EZA** (tan) within VchCAβ active site. H-bonds, and π-π stacking interactions are depicted as black, and cyan dashed lines, respectively. Clash contacts between the ligand-target surfaces are represented as orange clouds.

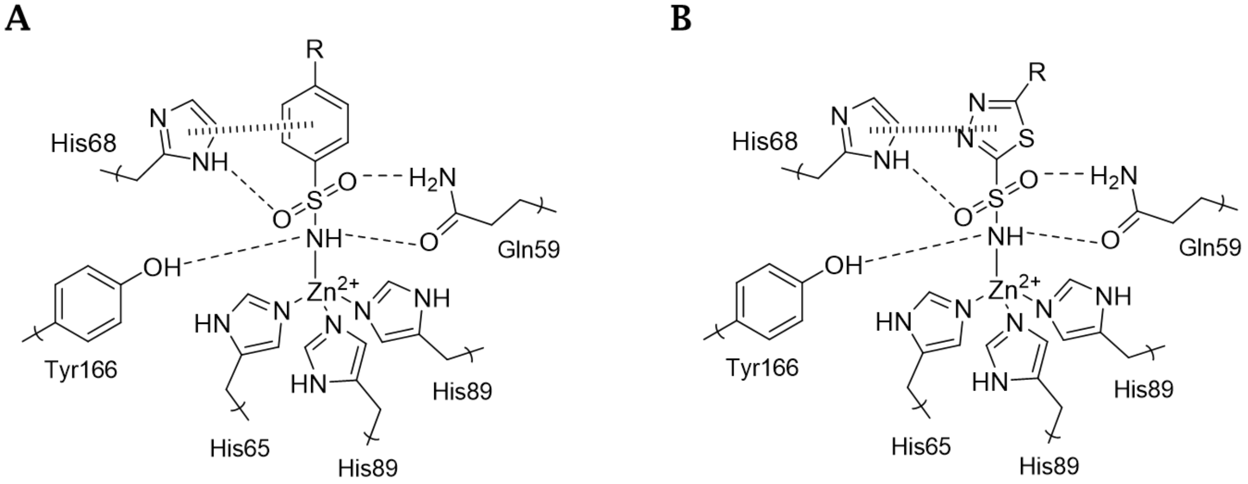

Figure S8. 2D representation of residues involved in the stabilization of (A) benzenesulfonamide and (B) 1,3,4-thiadiazole-2-sulfonamide inhibitors in the VchCAγ active site.

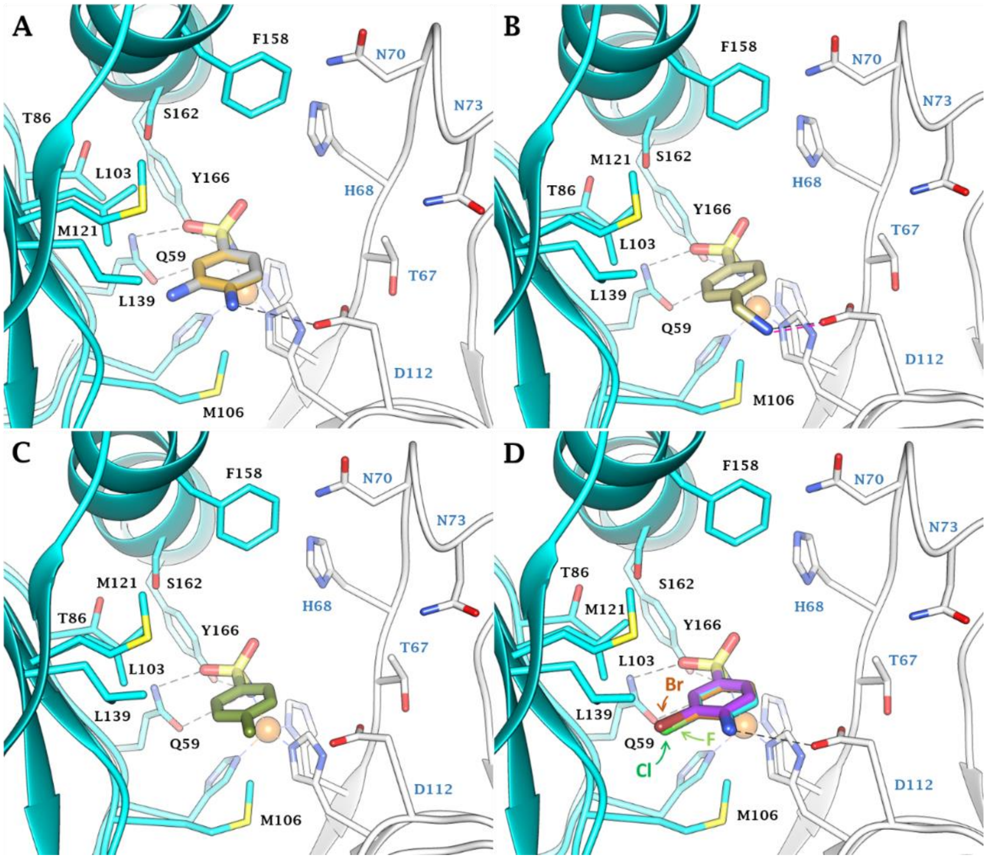

Figure S9. Predicted binding mode of ligands (A) **1** (light gray), and **2** (goldenrod), (B) **3** (dark khaki), (C) **6** (olive drab), (D) **7** (orange), **8** (cyan), and **9** (purple) within VchCAγ active site. H-bonds and salt bridge interactions are depicted as black and magenta dashed lines, respectively.

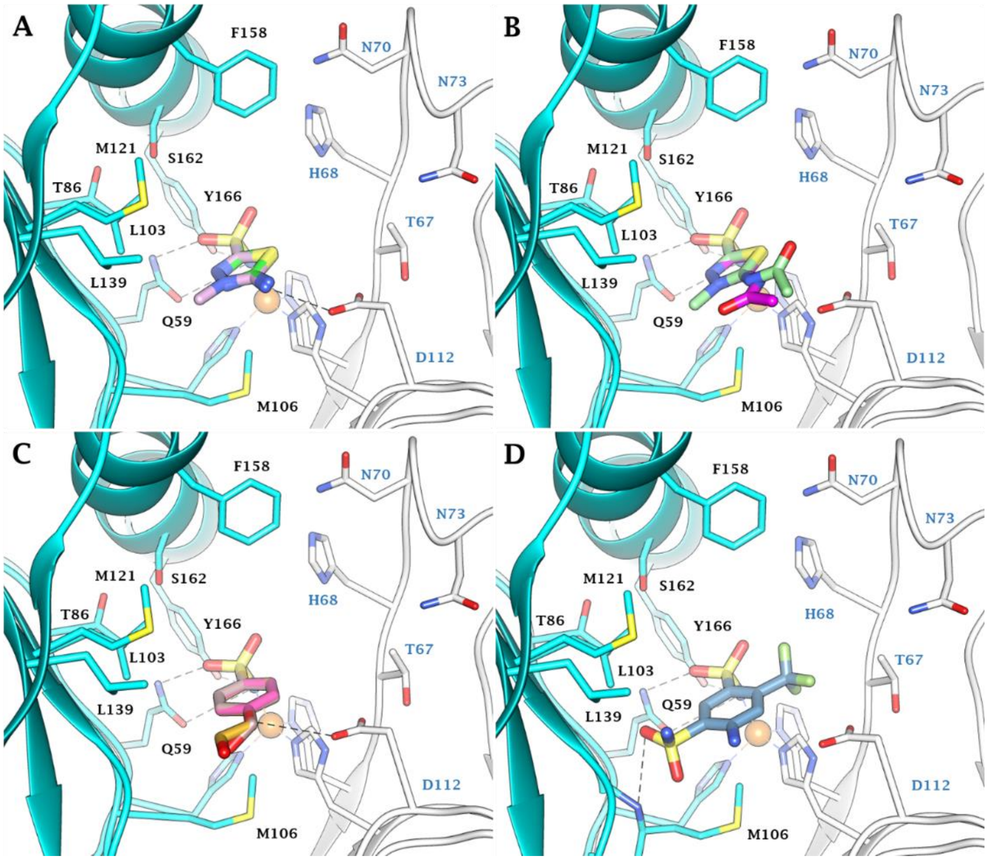

Figure S10. Predicted binding mode of ligands (A) **13** (green), and **14** (plum), (B) **AAZ** (magenta), and **MZA** (light green), (C) **15** (pink), **16** (rosy brown), and **17** (gold), and (D) **11** (steel blue) within VchCAγ active site. H-bonds are depicted as black dashed lines.

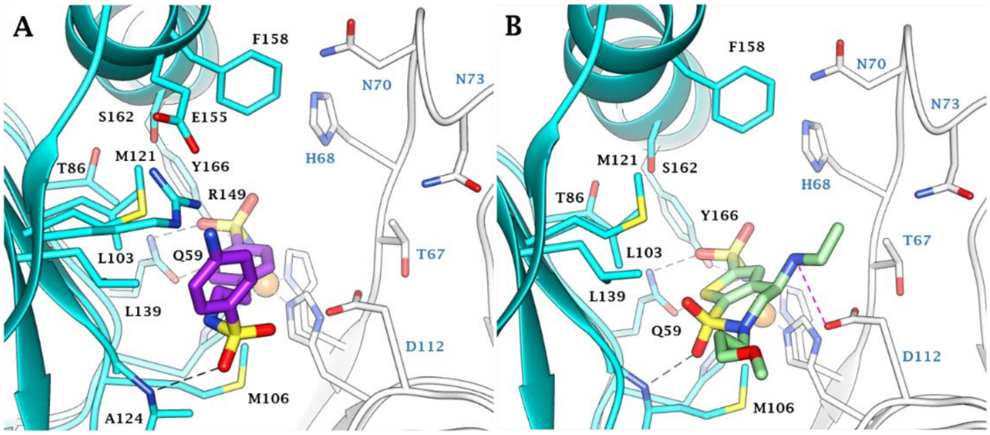

Figure S11. Predicted binding mode of ligands (A) **24** (purple), and (B) **BRZ** (light green) in VchCAγ active site. H-bonds and salt bridge interactions are depicted as black and magenta dashed lines, respectively.
